# A prospective cohort study on active surveillance after neoadjuvant chemoradiotherapy for esophageal cancer: protocol of Surgery As Needed for Oesophageal cancer-2

**DOI:** 10.1186/s12885-023-10747-z

**Published:** 2023-04-10

**Authors:** Charlène J. van der Zijden, Sjoerd M. Lagarde, Merel Hermus, Leonieke W. Kranenburg, J. Jan B. van Lanschot, Bianca Mostert, Joost J. M. E. Nuyttens, Lindsey Oudijk, Pieter C. van der Sluis, Manon C. W. Spaander, Maria J. Valkema, Roelf Valkema, Bas P. L. Wijnhoven, SANO-2 study group, Jan Willem T. Dekker, Willem E. Fiets, Hendrik H. Hartgrink, Wouter L. Hazen, Ewout A. Kouwenhoven, Grard A. P. Nieuwenhuijzen, Camiel Rosman, Johanna W. van Sandick, Meindert N. Sosef, Edwin S. van der Zaag

**Affiliations:** 1 Department of Surgery, Erasmus MC Cancer Institute, Erasmus University Medical Center, Dr. Molewaterplein 40, 3015GD Rotterdam, the Netherlands; 2 Department of Medical Psychology and Psychotherapy, Erasmus University Medical Center, Rotterdam, The Netherlands; 3 Department of Medical Oncology, Erasmus University Medical Center, Rotterdam, the Netherlands; 4 Department of Radiation Oncology, Erasmus University Medical Center, Rotterdam, the Netherlands; 5 Department of Pathology, Erasmus University Medical Center, Rotterdam, the Netherlands; 6 Department of Gastroenterology and Hepatology, Erasmus University Medical Center, Rotterdam, The Netherlands; 7 Department of Nuclear Medicine, Erasmus University Medical Center, Rotterdam, the Netherlands

**Keywords:** Esophageal cancer, Esophagogastric junction cancer, Chemoradiotherapy, Active surveillance, Standard esophagectomy, Decision counselling

## Abstract

**Background:**

Neoadjuvant chemoradiotherapy (nCRT) followed by esophagectomy is a standard treatment for potentially curable esophageal cancer. Active surveillance in patients with a clinically complete response (cCR) 12 weeks after nCRT is regarded as possible alternative to standard surgery. The aim of this study is to monitor the safety, adherence and effectiveness of active surveillance in patients outside a randomized trial.

**Methods:**

This nationwide prospective cohort study aims to accrue operable patients with non-metastatic histologically proven adenocarcinoma or squamous cell carcinoma of the esophagus or esophagogastric junction. Patients receive nCRT and response evaluation consists of upper endoscopy with bite-on-bite biopsies, endoscopic ultrasonography plus fine-needle aspiration of suspicious lymph nodes and ^18^F-fluorodeoxyglucose positron emission tomography/computed tomography scan. When residue or regrowth of tumor in the absence of distant metastases is detected, surgical resection is advised. Patients with cCR after nCRT are suitable to undergo active surveillance. Patients can consult an independent physician or psychologist to support decision-making. Primary endpoint is the number and severity of adverse events in patients with cCR undergoing active surveillance, defined as complications from response evaluations, delayed surgery and the development of distant metastases. Secondary endpoints include timing and quality of diagnostic modalities, overall survival, progression-free survival, fear of cancer recurrence and decisional regret.

**Discussion:**

Active surveillance after nCRT may be an alternative to standard surgery in patients with esophageal cancer. Similar to organ-sparing approaches applied in other cancer types, the safety and efficacy of active surveillance needs monitoring before data from randomized trials are available.

**Trial registration:**

The SANO-2 study has been registered at ClinicalTrials.gov as NCT04886635 (May 14, 2021) – Retrospectively registered.

## Background

Since the ChemoRadiotherapy for Oesophageal cancer followed by Surgery Study (CROSS) showed an improved 5-year overall survival after neoadjuvant chemoradiotherapy (nCRT) plus surgery compared to surgery alone, trimodality treatment has become a standard of care for resectable esophageal cancer [1–3]. The CROSS trial also showed that nearly a third of patients had a pathologically complete response (pCR) [1]. Estimated 5-year survival in patients with pCR is between 70 and 80% [4]. However, surgery likely has not contributed to this favorable outcome since this improved survival can be attributed to the introduction of neoadjuvant chemoradiotherapy [1]. Active surveillance could therefore be considered as an alternative treatment option in patients with a clinically complete response (cCR) after nCRT. To assess whether patients achieve cCR, response evaluations are performed consisting of ^18^F-fluorodeoxyglucose positron emission tomography/computed tomography (FDG-PET/CT) scan, upper endoscopy with bite-on-bite biopsies, and endoscopic ultrasound with fine-needle aspiration (EUS-FNA) of suspicious lymph nodes. Only when locoregional regrowth of cancer is (cyto)histologically proven or is highly suspected in the absence of distant metastases, patients are offered surgical resection.

The benefit of active surveillance might be that patients will not need esophagectomy in case of persistent cCR after nCRT. Esophagectomy is associated with substantial morbidity and has a lasting impact on patients’ health-related quality of life [5–7]. Also, patients who develop (early) distant metastases despite locoregional control of the disease are spared futile surgery. Hence, some 50% of patients still develop distant metastases despite radical surgery of which 75% of these within 2 years after esophagectomy. This underlines the current shortcomings of proper patient selection for curative treatment.

On the other hand, active surveillance has risks. Regrowth of tumor might remain undetected despite clinical response evaluations. This could lead to a non-resectable tumor or the development of metastases from residual tumor cells. Furthermore, delayed surgery might be associated with an increased postoperative morbidity [8].

Active surveillance or watchful waiting is already being applied in patients with rectal cancer. According to the 2019 Dutch guideline on colorectal cancer, watchful waiting should be discussed as an alternative to surgery in patients with cCR after nCRT [9]. However, implementation of watchful waiting instead of standard surgery after nCRT for rectal cancer is not supported by data from randomized trials, but based on data from observational studies including a multicenter registration study [10]. In esophageal cancer, randomized trials are still needed to show the safety and efficacy of active surveillance. The Surgery As Needed for Oesophageal Cancer (SANO) trial was initiated late in 2017 [11, 12]. The SANO trial was designed to test non-inferiority of active surveillance compared to standard esophagectomy after nCRT, and randomized hospitals to one of the two strategies in a cluster-randomized design. Inclusion of patients with cCR was finished mid-2021 and the primary endpoint (*i.e.* overall survival after at least two years) will be available end 2023. In France as well, a study on active surveillance is performed (Esostrate trial) in patients with epidermoid carcinoma or adenocarcinoma of the esophagus [13].

Several observations have been made during the SANO trial supporting the willingness of patients to opt for active surveillance. In the hospitals randomized to standard surgery, 25% of patients who obtained cCR refused an operation and requested active surveillance instead. In the hospitals randomized to active surveillance, less than 1% of patients switched to standard surgery. This is in line with a previous discrete choice study in which patients were willing to trade off 15% chance of being cured at 5 years after nCRT in order to circumvent surgery [14]. Furthermore, a recent individual patient-data meta-analysis showed comparable overall survival between active surveillance and standard esophagectomy in patients with locally advanced esophageal cancer [15]. The Dutch patient federation for cancer of the digestive tract is also closely involved and supports active surveillance.

Given these observations, active surveillance may be offered as an alternative to standard surgery after nCRT whilst patients are registered in a prospective study setting and the compliance to the active surveillance protocol is continuously monitored. The aim of the SANO-2 study is to monitor safety, adherence and effectiveness of active surveillance in patients that opt for active surveillance before the results of randomized studies become available.

## Methods

### Study design and recruitment

The SANO-2 study is a multicenter prospective observational extension study. The study is initiated by the Erasmus MC Cancer Institute and will be conducted at 11 of the 12 hospitals that participated in the SANO trial. The study has been approved by the local Ethics Committee (Erasmus University Medical Centre Rotterdam; MEC 2021–0068) and has been registered at ClinicalTrials.gov (NCT04886635) [16]. All patients will provide written informed consent prior to the start of the study. The study will be performed in accordance with the Declaration of Helsinki (64^th^ World Medical Association General Assembly, Fortaleza, Brazil, October 2013).

### Patients

Similar to the SANO trial, operable patients of 18 years or older who will undergo nCRT according to the CROSS regimen for histologically proven esophageal or junctional adenocarcinoma or squamous cell carcinoma are eligible. The primary tumor should be potentially resectable (cT1b-4aN0-3M0) based on staging examinations including upper endoscopy, EUS-FNA and FDG-PET/CT. Patients will be excluded when they meet one or more of the following criteria: non-FDG avid tumor at baseline; no definitive histology at baseline; initial treatment of cancer with endoscopic resection; underwent or planned for definitive chemoradiotherapy; linguistic or mental inability to understand the study; dementia or altered mental status prohibiting the understanding of and giving informed consent.

### Recruitment

All patients should be discussed in a multidisciplinary tumor board. If the board concludes that nCRT plus surgery is the preferred treatment, the attending surgeon will inform patients about the possibility of active surveillance within the SANO-2 study. Patients will receive a patient information letter. Patients who prefer standard esophagectomy, definitive chemoradiotherapy or patients who never want surgery at all will not be included in the study.

### Study procedures

**Fig. 1:**
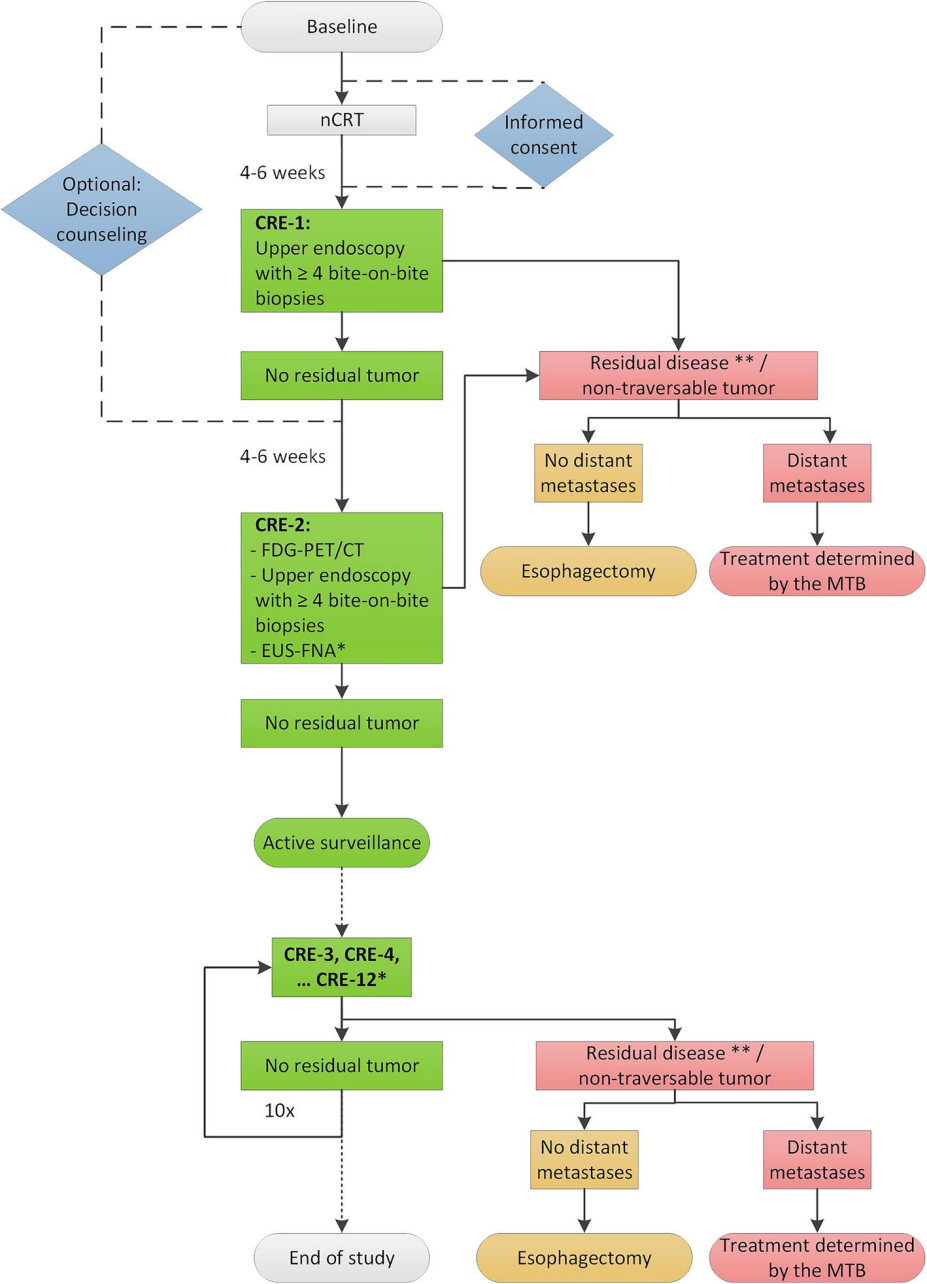
Flowchart of the SANO-2 study. nCRT: neoadjuvant chemoradiotherapy; CRE: clinical response evaluation; FDG-PET/CT: positron emission tomography-computed tomography; EUS: endoscopic ultrasound; FNA: fine-needle aspiration; MTB: multidisciplinary tumor board. *If FNA cytology obtained from suspicious lymph nodes is non-representative, EUS-FNA should be repeated within 2 weeks. If cytology of suspicious lymph nodes is non-representative again the patient should be considered to have residual disease. **Surgical resection can be offered to selected patients that have a high clinical/diagnostic suspicion of tumor regrowth, despite repeatedly negative (cyto)histology

The flowchart of the study is shown in Fig. 1.

#### Neoadjuvant chemoradiotherapy

The regimen consists of five weekly cycles of carboplatin intravenously at an area under the curve (AUC) of 2 mg/ml/min and paclitaxel intravenously at dose 50 mg/m^2^ on the first day of each week, with concurrent radiotherapy 41.4 Gy given in 23 fractions of 1.8 Gy for five days per week, starting on the first day of each cycle of chemotherapy [1].

#### CRE-1

The first clinical response evaluation (CRE-1) is performed 4–6 weeks after completion of nCRT. CRE-1 consists of an upper endoscopy with at least 4 bite-on-bite biopsies of the primary tumor location and of any areas suspect for residual disease. When taking bite-one-bite biopsies, the second biopsy is taken from the same location as the first biopsy [17]. Bite-on-bite biopsies are expected to increase the probability of detecting submucosal tumor deposits. In case of histologically proven residual tumor, high-grade dysplasia (as confirmed by two pathologists independently) or an endoscopically non-traversable tumor, the patient will undergo an FDG-PET/CT to exclude distant metastases and esophagectomy is advised. In case no residual tumor is detected, the patient will undergo a second clinical response evaluation (CRE-2).

#### CRE-2

CRE-2 is performed 10–12 weeks after completion of nCRT. CRE-2 consists of an FDG-PET/CT scan, and in case of no systemic disease, upper endoscopy with bite-on-bite biopsies and EUS-FNA of suspicious lymph nodes based on FDG-PET/CT and/or EUS will be performed. If locoregional residual disease is proven or highly suspected (including a non-traversable tumor, high-grade dysplasia or dubious cytology results after FNA of suspicious lymph nodes) and when there are no distant metastases, patients are advised to undergo surgical resection. If FNA-cytology obtained from suspicious lymph nodes is non-representative, EUS-FNA should be repeated within 2 weeks. If cytology of suspicious lymph nodes is non-representative again, the patient should be considered to have residual disease and surgical resection is advised. Endoscopic suspicion of residual tumor after nCRT is associated with presence of residual disease (positive predictive value 91%) and further treatment of these patients should therefore be discussed in a multidisciplinary team [18].

#### Active surveillance

Patients with cCR at CRE-2 are included in an active surveillance program. During active surveillance, patients will undergo CREs according to the SANO protocol [11, 12]. Each CRE consists of an FDG-PET/CT-scan, upper endoscopy with bite-on-bite biopsies and EUS-FNA of suspicious lymph nodes. CREs are scheduled every 3 months in the first year, every 4 months in the second year, every 6 months in the third year and yearly in the fourth and fifth year of follow up, or when symptoms or results of any diagnostic test require shorter assessment intervals such as increasing FDG uptake at the primary tumor site or dubious biopsy or cytology results. Surgical resection will be offered only to patients in whom locoregional regrowth is highly suspected or proven, without any signs of distant dissemination.

#### Decision counselling

An independent medical doctor or psychologist will be trained to elicit, explore and discuss patients’ preferences towards active surveillance or standard surgery. At the outpatient clinic, the patient is offered the possibility of decision counselling, either in person or by telephone or using video connection.

#### Surgery

Patients are offered surgical resection when locoregional residual disease is confirmed or highly suspected (*i.e.* high-grade dysplasia), or when there is a non-traversable tumor. Esophagectomy will be performed using a transthoracic or transhiatal approach. Open, hybrid and totally minimally invasive techniques can be used including robot-assisted approaches. Outcomes after surgery including pathology results are registered for all patients in the national Dutch Upper gastro-intestinal Cancer Audit (DUCA).

#### Pathology

All biopsies taken at CREs will be assessed by a specialized gastro-intestinal pathologist. Surgical resection is advised when vital tumor cells or high-grade dysplasia is detected. A finding of high-grade dysplasia should be confirmed by a second pathologist independently. If no malignancy is detected in the primary analysis of biopsies, deeper sections will be performed. If there is any doubt about the interpretation of the CRE biopsies, a second independent pathologist will be asked in consultation. All resection specimens will be assessed using the 8th edition of the UICC TNM cancer staging [19]. Tumor regression grade (TRG) will be determined according to the modified Mandard classification [20]. Patients will be offered nivolumab after esophagectomy according to standard of care when residual disease is present in the resection specimen [21].

#### Quality of life assessments

Data on quality of life will be retrieved from the Prospective Observational Cohort study of Oesophageal-gastric cancer Patients (POCOP; nationwide quality of life registry) by each participating center [22]. The Cancer Worry Scale and the Decisional Regret Scale will be used additionally to investigate concerns, fear of cancer recurrence and decisional regret and will be distributed at every CRE beyond CRE-2 [23, 24].

#### Data monitoring

The study coordinator of the Erasmus MC Cancer Institute will monitor adherence to the study protocol via remote access to patient data in the participating SANO-2 centers. Case report forms will be used to collect data on upper endoscopy (i.e. number and type of biopsies), EUS-FNA (i.e. if FNA is performed in case of suspicious lymph nodes) and FDG-PET/CT. All case report forms are stored centrally in the Castor EDC database.

## Study endpoints

The primary endpoint of the SANO-2 study is safety of active surveillance (including delayed surgery), defined by the number of patients with an adverse event. Adverse events are defined as complications from upper endoscopy with biopsies, EUS-FNA and FDG-PET/CT during active surveillance. Adverse events also include unresectable or incurable tumor (cT4b or M1), microscopically non-radical (R1) resection, postoperative mortality (90-day or in-hospital mortality), postoperative hospital stay of > 60 days, postoperative complications as defined by the Esophagectomy Complications Consensus Group (ECCG) and the development of distant metastases [25]. These data are compared with data from the DUCA.

Secondary endpoints are adherence to the study protocol and effectiveness of active surveillance, including overall survival and progression-free survival (Table 1). These endpoints also include the proportion of patients that opted for decision counselling as well as the proportion of patients who switched from active surveillance to standard surgery or vice versa; fear of recurrence of cancer (as assessed with the validated Cancer Worry Scale) and regret of the decision to undergo either active surveillance or surgery (as measured by the validated Decision Regret Scale) [23, 24].

**Table 1:** Overview of the secondary endpoints in adherence to the SANO-2 study protocol

Secondary endpoints
**Adherence and quality of active surveillance according to the protocol, including:**
⦁ The proportion of patients who meet all eligibility criteria;
⦁ The proportion of appropriately timed diagnostic modalities (according to timing of CREs in Fig. 1);
⦁ The proportion of CREs that are performed in correct order (FDG-PET/CT one week prior to combined upper endoscopy and EUS);
⦁ The proportion of upper endoscopies with at least 4 bite-on-bite biopsies or regular biopsies taken;
⦁ The proportion of FNA performed in case of suspicious lymph nodes;
⦁ The proportion of complete endoscopic reports (anatomic landmarks should be described in cm from incisors, including upper and lower tumor boundary, upper esophageal sphincter, Z-line (*i.e.* squamo-columnar junction), esophagogastric junction (*i.e.* upper border of gastric folds) and diaphragm impression);
⦁ The number of biopsies taken and quality of the biopsies, defined as (non-)representative by the pathologist
**Effectiveness of active surveillance outside the SANO trial, regarding the following endpoints:**
⦁ Rate of distant relapse, defined as the proportion of all patients with cCR who develop distant metastases;
⦁ Rate of locoregional relapse, defined as the proportion of patients with cCR who develop locoregional relapse;
⦁ Progression-free survival (PFS), defined as the interval between cCR and the earliest occurrence of disease progression resulting in primarily (or peroperatively) unresectable disease, locoregional regrowth (after completion of therapy), distant dissemination (during or after completion of treatment) or all-cause death;
⦁ Overall survival (OS) of patients with cCR at CRE-2 (*i.e.* 10–12 weeks after completion of nCRT), defined from date of diagnosis to date of all-cause death or to last day of follow-up

## Patient safety

The SANO-2 study has no data safety monitoring board (DSMB) as it is an observational study. However, the safety and feasibility of active surveillance are periodically monitored by a DSMB in the SANO trial, including the following parameters:

1. Timely detection of resectable locoregional regrowth (< T4b) in the active surveillance arm;
2. Feasibility of achieving a radical resection (R0) in the active surveillance arm;
3. Acceptable postoperative morbidity for delayed surgery in the active surveillance arm;
4. Acceptable distant dissemination rate in the active surveillance arm (as compared to the immediate surgery arm).

## Multidisciplinary meetings

To monitor the inclusion of patients in the SANO-2 study in participating centers and to discuss clinical dilemma’s from daily practice, a multidisciplinary meeting with the participating centers will be organized several times per year. The aim is to optimize the active surveillance strategy and to share difficult cases while implementing active surveillance in daily clinical practice.

## Statistical analysis

### Sample size calculation

Since this is an observational study, a power calculation is not applicable. All patients who meet the eligibility criteria can be included for the duration of the SANO-2 study.

#### Data analysis

The type, number and severity of adverse events during the registration of patients in active surveillance will be extracted from patient records. Descriptive statistics will include median and interquartile range or mean and standard deviation for continuous variables, and frequency counts with percentages for categorical variables. Proportions and rates will be described with descriptive statistics. Overall survival will be calculated using the Kaplan–Meier method. Cumulative incidence functions will be used to illustrate absolute risks of locoregional and distant relapse. Fear of cancer recurrence and decisional regret will be scored according to the Cancer Worry Scale and Decision Regret Scale scoring manuals respectively [23, 24].

## Discussion

SANO-2 is a prospective observational extension study, investigating the safety, adherence and effectiveness of active surveillance in patients with resectable esophageal cancer or cancer of the EGJ.

Offering active surveillance to patients after nCRT as an alternative to standard surgery while awaiting safety and efficacy data from randomized phase-III trials can be debated. A meta-analysis of 7 cohort studies showed that overall survival between patients undergoing standard surgery or active surveillance after nCRT is comparable [15]. However, this study has several possible confounders. The study included predominantly retrospective studies with a small sample size. Patients included in active surveillance were highly selected and possibly represent a group with a more favorable prognosis. In addition, the standard surgery group did not include patients who refused surgery or were unfit for surgery which, despite propensity score matching, results in substantial selection bias. Also, the rate and timing of distant metastases were not sufficiently reported for a reliable comparison between the groups, and diagnostic tests used in response evaluations differed between the studies.

The Dutch patient federation for cancer of the digestive tract states that active surveillance for esophageal cancer should be discussed with patients while awaiting SANO results, as there was equipoise for active surveillance in a randomized study design. Secondly, there have been no safety issues reported so far and patients should be given the opportunity to make a well-informed shared decision for active surveillance based on the currently available data. The potential risks associated with active surveillance are regrowth of tumor that may be detected beyond the resectability limit, development of distant metastases from residual disease and a possible increase in postoperative morbidity of delayed surgery. The meta-analysis reported that 39% of patients in the active surveillance group developed locoregional recurrence after a median follow-up of 50 months of whom seven patients (7.5%) had synchronous distant metastases. Importantly, a radical resection rate (R0) of 95% was reached in patients undergoing postponed esophagectomy [15].

To ensure that tumor regrowth after nCRT will be detected at a curable stage, surveillance is performed. The majority of locoregional regrowths are expected to occur within 12 months after nCRT, and nearly all within 24 months after nCRT. For now, the SANO protocol stipulates that all patients will undergo response evaluations for 5 years. It may well be that the frequency and duration of clinical response evaluations could be tailored according to histological tumor type, since esophageal squamous cell carcinomas are more sensitive to nCRT than adenocarcinomas leading to a higher percentage of pCR. However, thus far it remains unknown if the chance for regrowth of cancer is also lower in patients with squamous cell cancer. It is also important to assess patient’s wishes regarding continuation of active surveillance and whether there is a disproportionate recurring fear for the outcome of the response evaluations, which will be measured using the Cancer Worry Scale [24]. Also, indications for surgery may expand. For example, in patients with oligometastatic disease there may be room for curative treatment options including esophagectomy as part of an intense multimodality treatment [26].

The present study protocol defines that surgical resection should also be offered to patients with suspected locoregional regrowth to minimize the risk that clinically suspicious lesions with indefinite histological proof advance to an incurable disease stage. It may be difficult for the pathologist to detect viable cancer cells in a biopsy specimen given the irradiated tumor with inflammatory and reactive changes. Clinical response evaluations are not infallible. Data from the preSANO trial showed false-negative results of endoscopy with bite-on-bite biopsies and EUS-FNA in 10% [17]. Therefore, in case of suspicion of residual tumor or high-grade dysplasia, immediate surgery is advised. In patients with an esophagus stricture and a non-traversable tumor on upper endoscopy, accurate surveillance of the tumor bed is impossible and therefore patients are advised to undergo surgery. So far, approximately half of the patients with a non-traversable tumor on upper endoscopy have residual tumor in the resection specimen (preliminary data from the SANO trial and SANO-2 study). Data from the SANO trial and the present study will hopefully supply more information on these important considerations, and this will affect implementation of active surveillance in standard practice when non-inferiority is proven.

Making a well-informed decision between an ‘experimental treatment’ and ‘standard treatment’ is difficult for patients in clinical practice [27]. Some patients feel choosing active surveillance as ‘giving up’ or may feel that the stress associated with frequent CREs may not outweigh the likelihood that surgery can be omitted. Decision counselling is a tool to elicit, explore and discuss patients’ preferences in such a way, that patients are enabled to reflect on all aspects of their preference. It is not the purpose of this consultation to make patients change their mind, but rather to ensure that they have had the opportunity to consider pre-established and well-balanced information on both treatments options and are challenged to thoroughly consider the consequences of each option for them personally. Decision counselling is included in the SANO-2 study to give insight into the patient’s information needs and allows us to develop decision aids. This provides an opportunity for clinicians to gain experience of how active surveillance can be discussed as alternative treatment option in clinical practice. Standardizing patient information on a national level will increase patients’ empowerment, enhance shared decision making and reduce interhospital variation.

The term ‘active surveillance’ reflects the use of response evaluations to detect regrowth of cancer. For rectal cancer or prostate cancer, definitions such as ‘wait and see’ or ‘watchful waiting’ are frequently used. The term ‘watchful waiting’ gives the impression that less intense monitoring of tumor regrowth is used. Similar to a recent consensus statement in prostate cancer by international experts, it will also be helpful to obtain unambiguous use of semantics in active surveillance for esophageal cancer [28]. Watchful waiting with selective delayed intervention is already being used in several other cancers including rectal cancer and prostate cancer [9, 29]. For rectal cancer, watchful waiting was applied in a few highly specialized centers within the framework of clinical trials. The application of watchful waiting is currently being expanded worldwide, whereby patients can be registered and monitored in an international database to ensure a certain level of quality control. A recent analysis of this registry showed no difference in 5-year survival and distant dissemination rate between patients undergoing watchful waiting or standard resection [10]. In contrast to active surveillance in esophageal cancer, watchful waiting in rectal cancer patients is recommended as alternative treatment option according to current guidelines and it is already offered outside a registered study context. To draw a parallel, pCR rate after nCRT in rectal cancer is comparable (27%) to esophageal cancer (25–30%) [1, 30, 31]. Also, the risk of local regrowth is comparable for both tumor types estimated to be 7–33% within 3 years in rectal cancer [32–34] and around 30% in esophageal cancer (preliminary data from the SANO trial and SANO-2 study). The combined diagnostic tests used in the response evaluation of rectal cancer (MRI, endorectal ultrasonography and CT) have a negative predictive value (NPV) ranging from 42–53% [35]. In esophageal cancer, detection of regrowth with upper endoscopy, EUS and FDG-PET/CT has a NPV ranging from 35–44% [17]. Rectal cancer surgery is associated with a 31% rate of major complications, approximately 25% of patients have a permanent stoma and anorectal and sexual dysfunction is seen in more than 60% of patients [36–38]. After esophagectomy, over 60% of patients get a complication with 25% classified as major and the 30-day mortality rate is around 3%. Esophagectomy has a great impact on patient’s quality of life lasting for years after surgery [1]. The morbidity associated with surgery for both tumor types supports the exploration and implementation for less invasive treatments. Although watchful waiting is already being applied in patients with rectal cancer, active surveillance in patients with esophageal cancer is still under investigation and should be carefully evaluated before implementation on a wider scale.

The SANO-2 study offers a platform to assess the quality of active surveillance by registration of CREs, treatment-related complications and survival. Multidisciplinary meetings with participating centers are organized to discuss possible safety issues and learn from experience. Participating centers receive feedback on the completeness and quality of clinical response evaluations and outcomes including patient-reported quality of life.

## Data Availability

Not applicable, since this is a study protocol.

## Abbreviations

AUC: Area Under the Curve
cCR: Clinically Complete Response
CRE: Clinical Response Evaluation
CROSS: ChemoRadiotherapy for Oesophageal cancer followed by Surgery Study
CT: Computed Tomography
DSMB: Data Safety Monitoring Board
DUCA: Dutch Upper gastro-intestinal Cancer Audit
ECCG: Esophagectomy Complications Consensus Group
EDC: Electronic Data Capture
EGJ: EsophagoGastric Junction
EUS: Endoscopic UltraSonography
^18^F-FDG: ^18^F-fluorodeoxyglucose
FNA: Fine Needle Aspiration
Gy: Gray
MC: Medical Center
MRI: Magnetic Resonance Imaging
MTB: Multidisciplinary Tumor Board
nCRT: Neoadjuvant ChemoRadioTherapy
NPV: Negative Predictive Value
OS: Overall Survival
pCR: Pathologically Complete Response
PET/CT: Positron Emission Tomography/Computed Tomography
PFS: Progression-Free Survival
POCOP: Prospective Observational Cohort study of Oesophageal-gastric cancer Patients
SANO: Surgery As Needed for Oesophageal cancer
TNM: Tumor Node Metastasis classification system
UICC: Union for International Cancer Control
